# Application of Fourier transform infrared (FT-IR) spectroscopy to discriminate serotypes of *Streptococcus suis*

**DOI:** 10.1186/s13567-026-01870-3

**Published:** 2026-10-31

**Authors:** Francesca Romana Massacci, Miriam Cordovana, Elisa Albini, Lucilla Cucco, Francesca Piersanti, Elisa Russo, Patrick Leo, Andrea Baldaccini, Andrea Luppi, Denis Vio, Marta Paniccià, Giovanni Pezzotti, Chiara Francesca Magistrali

**Affiliations:** 1 Istituto Zooprofilattico Sperimentale dell’Umbria e delle Marche “Togo Rosati”, Via G. Salvemini 1, 06126 Perugia, Italy; 2 Bruker Daltonics GmbH and Co. KG, Bremen, Germany; 3 Istituto Zooprofilattico Sperimentale della Lombardia e dell’Emilia Romagna “Bruno Ubertini”, Via Bianchi, 9, 25124 Brescia, Italy; 4 Istituto Zooprofilattico Sperimentale delle Venezie, Viale dell’Università 10, 35020 Legnaro, Italy

**Keywords:** *Streptococcus suis*, Fourier transform infrared spectroscopy, FT-IR, serotypes

## Abstract

*Streptococcus suis* is a major swine pathogen and is considered an emerging zoonotic agent. Serotyping plays a crucial role for epidemiological investigations, pathogenicity assessment, and vaccine strategy. The present study evaluated the applicability of Fourier transform infrared (FT-IR) spectroscopy, using the IR Biotyper system, for the serotype discrimination of 94 *S. suis* isolates belonging to 13 distinct serotypes. Analysis of the 1300–800 cm^−1^ spectral region demonstrated the highest discriminatory power. Principal component analysis (PCA)/linear discriminant analysis (LDA) showed clear separation among the tested serotypes, except for serotypes 1/2 and 2. The classifier achieved overall accuracy of 90%, which increased to 96.7% when serotypes 2 and 1/2 were considered as a single category. These findings demonstrate that FT-IR spectroscopy represents a promising, rapid, and cost-effective tool for *S. suis* serotype discrimination. Its performance, ease of use, and compatibility with machine learning approaches support its potential implementation in routine diagnostic and epidemiological workflows. As previously reported for other clinically relevant bacteria, FT-IR spectroscopy may serve as a valuable complementary method to molecular typing, particularly in settings requiring rapid decision-making and high-throughput analysis.

**Supplementary Information:**

The online version contains supplementary material available at https://doi.org/10.1186/s13567-026-01870-3.

## Introduction

*Streptococcus suis* is an important pathogen in swine and an emerging zoonotic agent [1–4]. This bacterium is a natural inhabitant of the upper respiratory tract of pigs, and it is endemic in all pig-producing countries. In pigs, *S. suis* causes meningitis, septicaemia, polyserositis, arthritis, and endocarditis, mainly during the post-weaning period, and it is a source of concern for farmers due to potential economic losses and its impact on the welfare of infected pigs [2]. Human infection is acquired after occupational contact or via the ingestion of undercooked pork-derived products and is associated with meningitis, endocarditis, septicaemia, deafness, and death [5].

*Streptococcus suis* is a heterogeneous species. Until 2005, *S. suis* was divided into 35 serotypes (SS1–34 and 1/2), based on capsular polysaccharides, but six serotypes were recently reclassified as belonging to other *Streptococcus* species. Currently, there are 29 recognized *S. suis* serotypes (SS) [6, 7]. *Streptococcus suis* serotyping is commonly performed using a two-step multiplex polymerase chain reaction (PCR) targeting genes within the capsular polysaccharide locus. Although effective, this approach is time-consuming and requires a relatively long workflow. Most *S. suis* infections in humans and swine are caused by *S. suis* serotype 2 (SS2), but the predominant serotypes causing invasive disease in swine vary according to the time and region [8]. In some European countries, *S. suis* SS9 has emerged as the leading cause of invasive diseases in pigs [2, 8–10]. Most cases of swine disease in Italy are caused by serotypes 9 and 1/2, but other serotypes, such as serotype 7, are frequently identified, contributing to the considerable heterogeneity characteristic of this microbial species [11]. Serotypes other than SS2 are less frequently responsible for human cases [8]. Of note, despite the increased frequency of pig infections caused by SS9, the first human case of an SS9 infection was documented in Thailand in 2015 [12]. The identification of the serotype of *S. suis* is therefore relevant not only for epidemiological purposes but also for assessing the pathogenic and zoonotic potential of an isolate. The characterization of the serotype is also a prerequisite for vaccine selection. Indeed, the vaccines currently available in Europe are bacterin-based and do not guarantee cross-protection among different serotypes [13].

Fourier transform infrared (FT-IR) spectroscopy is a phenotypic method commonly employed in chemistry to determine the molecular composition of a wide range of sample types. The operational principle of FT-IR spectroscopy relies on the exploitation of distinct absorption characteristics exhibited by covalent bonds within different functional groups in the infrared region. This rapid, simple, inexpensive, and high-throughput technique has recently been successfully applied in various microbiology fields, yielding significant results and revolutionizing aspects of microbiological research and diagnostics [14]. The fundamental principle behind FT-IR spectroscopy lies in the interaction between infrared radiation and the vibrations of molecules. Each functional group within a molecule possesses a unique vibrational energy, and when infrared light of a matching frequency interacts with the molecule, it can cause changes in the molecule’s dipole moment, resulting in the absorption of the radiation [15]. This absorption pattern, analysed across a spectrum of infrared frequencies, generates a characteristic “fingerprint” that reflects a unique absorption profile. The combined spectral absorption of molecules like lipids, proteins, and carbohydrates produces a distinctive fingerprint for each microbial cell [16–18], a highly specific IR signature applicable for identification from the genus to the species level, and even down to serogroup/serotype and strain level [14, 16, 19–21]. Given the discriminatory capability comparable to molecular genetic methods, it has also been successfully used for management of nosocomial outbreaks and in routine surveillance for bacterial relatedness [17, 20, 22–25].

Comprehensive knowledge of circulating *S. suis* strains is essential for both epidemiological surveillance and disease control. The present study aimed to evaluate the applicability of the IR Biotyper (IRBT), a commercially available FT-IR spectroscopy-based system, for the discrimination of *S. suis* strains according to their serotype.

## Materials and methods

### Bacterial isolates

A total of 92 isolates of *S. suis* were analysed, plus one reference strain (ATCC43765). The isolates were collected from piglets affected by meningitis (63), pericarditis (1), arthritis (4), septicaemia (19), and pneumonia (4) (Table 1), and belonged to 13 serotypes and 23 sequence types (STs). The isolates originated from 11 Italian regions and 22 provinces, providing broad geographical coverage of the country, with several regions from Northern, Central, and Southern Italy represented (Table 1).

**Table 1:** **List of strains analysed in the study**

Isolates	Year	Organ of isolation	Country	Region	Province	Serotype	Sequence type	Published in
S121	2017	Brain	Italy	Emilia-Romagna	Reggio Emilia	23	108	Cucco et al. [11]
S126	2017	Brain	Italy	Lombardy	Mantova	2	ND	Present study
S127	2017	Brain	Italy	Lombardy	Brescia	1/2	1	Cucco et al. [11]
S129	2017	Brain	Italy	Piedmont	Cuneo	9	123	Massacci et al. [28]
S136	2017	ND	Italy	Piedmont	Cuneo	9	1541	Massacci et al. [28]
S138	2017	Heart	Italy	Lombardy	Mantova	1/2	1	Cucco et al. [11]
S145	2017	Brain	Italy	Lombardy	Mantova	4	1543	Cucco et al. [11]
S146	2017	Brain	Italy	Lombardy	Mantova	9	123	Massacci et al. [28]
S147	2017	Brain	Italy	Piedmont	Cuneo	1	11	Cucco et al. [11]
S148	2017	Brain	Italy	Piedmont	Cuneo	1/2	7	Cucco et al. [11]
S153	2018	Liver	Italy	ND	ND	2	1	Cucco et al. [11]
S159	2018	Brain	Italy	Piedmont	Cuneo	10	1547	Cucco et al. [11]
S161	2018	Kidney	Italy	Umbria	Perugia	1/2	1	Cucco et al. [11]
S163	2018	Kidney	Italy	Lombardy	Brescia	8	1545	Cucco et al. [11]
S164	2018	Brain	Italy	Piedmont	Cuneo	10	1547	Cucco et al. [11]
S165	2018	Brain	Italy	Lombardy	Mantova	1/2	1	Cucco et al. [11]
S166	2018	Brain	Italy	Emilia-Romagna	Reggio Emilia	15	1548	Cucco et al. [11]
S186	2018	Kidney	Italy	Marche	Ascoli Piceno	19	1549	Cucco et al. [11]
S225	2018	Brain	Italy	Lombardy	Mantova	2	7	Cucco et al. [11]
S241	2018	Spleen	Italy	Umbria	Perugia	9	123	Massacci et al. [28]
S250	2018	Kidney	Italy	Lombardy	Mantova	1/2	1	Cucco et al. [11]
S271	2018	Brain	Italy	Lombardy	Mantova	9	123	Massacci et al. [28]
S273	2018	Brain	Italy	Lombardy	Mantova	4	94	Cucco et al. [11]
S284	2018	Brain	Italy	Abruzzo	Chieti	1/2	7	Cucco et al. [11]
S299	2018	Brain	Italy	ND	ND	9	1540	Massacci et al. [28]
S302	2018	Brain	Italy	ND	ND	9	1540	Massacci et al. [28]
S304	2018	Lung	Italy	ND	ND	5	1544	Cucco et al. [11]
S306	2018	Brain	Italy	ND	ND	9	123	Massacci et al. [28]
S308	2018	Brain	Italy	Piedmont	Cuneo	10	1547	Cucco et al. [11]
S309	2018	Brain	Italy	ND	ND	9	123	Massacci et al. [28]
S311	2018	Kidney	Italy	ND	ND	4	94	Cucco et al. [11]
S312	2018	Kidney	Italy	ND	ND	8	1546	Cucco et al. [11]
S313	2018	Kidney	Italy	ND	ND	9	123	Massacci et al. [28]
S314	2018	Kidney	Italy	ND	ND	1/2	1	Cucco et al. [11]
S315	2018	Kidney	Italy	ND	ND	4	1544	Cucco et al. [11]
S317	2018	Kidney	Italy	ND	ND	1/2	1	Cucco et al. [11]
S318	2018	Brain	Italy	ND	ND	1/2	7	Cucco et al. [11]
S320	2018	Kidney	Italy	ND	ND	1/2	7	Cucco et al. [11]
S321	2018	Brain	Italy	Piedmont	Cuneo	10	1547	Cucco et al. [11]
S322	2018	Brain	Italy	Emilia-Romagna	Reggio Emilia	10	1547	Cucco et al. [11]
S323	2018	Brain	Italy	Piedmont	Asti	10	1547	Cucco et al. [11]
S324	2019	Joint	Italy	Marche	Macerata	1	11	Cucco et al. [11]
S33	2007	Lung	Italy	ND	ND	2	1	Present study
S336	2019	Brain	Italy	Umbria	Perugia	1/2	1	Cucco et al. [11]
S350	2019	Brain	Italy	Lombardy	Mantova	1/2	1	Cucco et al. [11]
S361	2019	Joint	Italy	Marche	Macerata	1/2	1	Cucco et al. [11]
S365	2019	Liver	Italy	Molise	Isernia	9	123	Massacci et al. [28]
S367	2019	Brain	Italy	Umbria	Perugia	2	ND	Present study
S368	2019	Brain	Italy	Umbria	Perugia	1/2	1	Cucco et al. [11]
S374	2019	Lung	Italy	Tuscany	Siena	2	7	Cucco et al. [11]
S408	2019	Brain	Italy	Umbria	Perugia	1/2	1	Cucco et al. [11]
S412	2019	Brain	Italy	Piedmont	Torino	9	123	Massacci et al. [28]
S414	2019	Brain	Italy	Piedmont	Cuneo	2	1	Cucco et al. [11]
S417	2019	Brain	Italy	Marche	Ancona	7	29	Cucco et al. [11]
S424	2019	Brain	Italy	Friuli-Venezia Giulia	Pordenone	2	7	Cucco et al. [11]
S425	2019	Brain	Italy	Friuli-Venezia Giulia	Gorizia	9	123	Massacci et al. [28]
S426	2019	Brain	Italy	Veneto	Treviso	9	123	Massacci et al. [28]
S427	2019	Brain	Italy	Veneto	Treviso	9	123	Massacci et al. [28]
S429	2019	Brain	Italy	Veneto	Padova	2	1	Cucco et al. [11]
S430	2019	Brain	Italy	Friuli-Venezia Giulia	Udine	9	94	Massacci et al. [28]
S431	2019	Brain	Italy	Friuli-Venezia Giulia	Pordenone	7	29	Cucco et al. [11]
S432	2019	Brain	Italy	Veneto	Treviso	7	29	Cucco et al. [11]
S435	2019	Brain	Italy	Veneto	Venezia	9	123	Massacci et al. [28]
S437	2019	Brain	Italy	Friuli-Venezia Giulia	Udine	9	123	Massacci et al. [28]
S438	2019	Brain	Italy	Marche	Ascoli Piceno	9	123	Massacci et al. [28]
S445	2020	ND	Italy	ND	ND	4	ND	Present study
S447	2020	Brain	Italy	Piedmont	Cuneo	9	1540	Massacci et al. [28]
S475	2020	Joint	Italy	Lombardy	Brescia	9	1953	Massacci et al. [28]
S483	2020	Brain	Italy	Marche	Ascoli Piceno	23	ND	Present study
S484	2020	Brain	Italy	Abruzzo	Chieti	8	3239	Present study
S485	2020	Brain	Italy	Lombardy	Cremona	9	1953	Massacci et al. [28]
S494	2020	Brain	Italy	Friuli-Venezia Giulia	Udine	9	123	Massacci et al. [28]
S495	2020	Brain	Italy	Friuli-Venezia Giulia	Udine	1–14	3242	Present study
S524	2020	Brain	Italy	Lombardy	Mantova	9	123	Massacci et al. [28]
S530	2020	Brain	Italy	Veneto	Padova	9	1546	Massacci et al. [28]
S541	2021	Joint	Italy	Basilicata	Potenza	1–14	ND	Present study
S547	2021	Brain	Italy	Veneto	Treviso	9	1540	Massacci et al. [28]
S548	2021	Brain	Italy	Veneto	Treviso	8	3244	Present study
S551	2021	Brain	Italy	Piedmont	Torino	9	1540	Massacci et al. [28]
S579	2005	Brain	Italy	Lombardy	Cremona	9	16	Massacci et al. [28]
S580	2002	Lung	Italy	Lombardy	Brescia	9	1956	Massacci et al. [28]
S584	2008	Spleen	Italy	Lombardy	Cremona	9	1957	Massacci et al. [28]
S593	2021	Brain	Italy	Friuli-Venezia Giulia	Udine	9	123	Massacci et al. [28]
S596	2021	Brain	Italy	Marche	Macerata	9	123	Massacci et al. [28]
S597	2021	Brain	Italy	Umbria	Perugia	9	16	Massacci et al. [28]
S598	2021	Liver	Italy	Umbria	Perugia	2	ND	Present study
S599	2021	Brain	Italy	Tuscany	Arezzo	9	16	Massacci et al. [28]
S600	2019	Brain	Italy	Friuli-Venezia Giulia	Pordenone	9	94	Massacci et al. [28]
S601	2019	Pericardium	Italy	Friuli-Venezia Giulia	Pordenone	9	94	Massacci et al. [28]
S602	2021	Spleen	Italy	Friuli-Venezia Giulia	Pordenone	9	94	Massacci et al. [28]
S684	2022	Brain	Italy	Abruzzo	Chieti	9	123	Present study
S694	2022	Brain	Italy	Piedmont	Torino	9	1540	Present study

*ND* not determined.

The identification of SS was performed using PCR-based serotyping methods [26, 27]. The majority of the isolates had also been subjected to whole-genome sequencing (WGS) as part of previous studies [11, 28]. For WGS-characterized isolates, in silico multi-locus sequence type (MLST) analysis was performed by submitting the sequences to the *S. suis* MLST database to determine allele profiles and assign sequence types (STs) [11, 28].

### Sample preparation

The samples were cultured on 5% sheep blood agar (Biolife Italiana S.r.l., Milan, Italy) incubated with 5% CO_2_ at 37 °C for 24 h. α-Haemolytic colonies were selected and confirmed as belonging to the *S. suis* species by matrix-assisted laser desorption ionization–time-of-flight mass spectrometry (MALDI-TOF MS) (MALDI Biotyper^®^, Bruker Daltonics GmbH, Bremen, Germany). FT-IR analysis was performed with the IR Biotyper^®^ system (IRBT, Bruker Daltonics). Sample preparation for IRBT analysis was performed according to the manufacturer’s instructions with the smear-ultraviolet (UV) protocol. Briefly, a 1-µl loop of bacterial cultures were collected and distributed on the whole spot of the 96-spot silicon IRBT sample plate. The samples were spotted in four technical replicates. A UV lamp was then used to disinfect clean benches at a distance between lamp and plate of approximately 10 cm, to irradiate the samples for at least 10 min. For each run, quality control was performed using the infrared test standards IRTS 1 and 2 provided in the IR Biotyper kit (Bruker Daltonics). All steps for IRBT sample preparation and measurements were carried out on a standard laboratory bench under ambient laboratory conditions without specific control of temperature or humidity.

### Spectra acquisition and data analysis

Spectra were acquired and processed with OPUS v8.2 and IR Biotyper^®^ client software v4.0 (Bruker Daltonics), using default settings recommended by the manufacturer. Spectra were acquired in the whole mid-IR range (4000–500 cm^−1^). After acquisition, the second derivative of the absorption values was calculated over nine data points using the Savtizky–Golay algorithm. The spectra were then vector-normalized and subsequently interpolated to have one data point for each integer wavenumber. Overall, 368 spectra were generated, and no spectra were excluded from the evaluation. The spectral regions corresponding to the absorption of infrared radiation by the main biological macromolecule classes (carbohydrates: 1200–900 cm^−1^; lipids: 3200–2800 cm^−1^ and 1500–1400 cm^−1^; proteins: 1800–1500 cm^−1^) were investigated by exploratory data analysis to find those that were most discriminatory. The spectral region of 1300–800 cm^−1^, set as default in the IR Biotyper software, was found to provide the best discriminatory power, and therefore it was used to perform similarity analysis by hierarchical cluster analysis (HCA), principal component analysis (PCA), and linear discriminant analysis (LDA), which are algorithms incorporated into the IR Biotyper^®^ software for automated application.

### Machine learning and development of automated classifiers

The artificial intelligence capabilities of the IRBT software were applied to develop a classifier as a proof of concept of the FT-IR-based capability to differentiate *S. suis* at the serotype level. Three different machine learning algorithms were tested: support vector machine with linear kernel (linear SVM), radial basis function support vector machine (RBF SVM), and artificial neural network (ANN). These well-established algorithms, which come from openly accessible Java libraries, are incorporated into the IR Biotyper software. For each one, only a few parameters can be set by the users [cost factor (C) for both linear and RBF SVM, and number of training cycles for ANN].

As a general principle, the machine learning algorithms included in the IRBT software are trained with a set of well-characterized isolates belonging to the classes of interest, to “learn” to recognize the specific characteristics of each class (in this case the serotypes of *S. suis* isolates). A cross-validation (four-fold or leave-one-isolate-out) is then performed to verify the predictive accuracy on the training set. The classification process relies on the attribution of one of the classes included in the training dataset to unknown samples subjected to the analysis, according to the model calculated by the algorithm. The classification result is delivered with a “traffic light” colour code scoring system (green–yellow–red), which is related to the reliability of the classification. The threshold values applied to define the three categories were extrapolated from the distribution of the outlier values of the validation cohort of samples considering the Youden index. A “green score” result means that the sample spectrum is located within the spectral space of the training set (high reliability). A “yellow score” result means that the sample spectrum is located at the periphery of the spectral space of the training set (moderately reliable). A “red score” value means that the sample spectrum is located far away from the spectra of any class included in the training set; therefore, it cannot be considered reliable.

In this study, the application of machine learning to create a classifier was intended as a proof of concept to assess the applicability of FT-IR spectroscopy for the typing of *S. suis* at the serotype level using an automated approach, which could pave the way for the development of a general and universal classifier for routine use. Given the unbalanced distribution of the isolates in terms of serotypes, the whole dataset was used to train the classifier. Four-fold cross-validation was performed to verify the predictive accuracy on the training set. In this cross-validation, the folds were created randomly, and the spectra of the same isolate could have been split between training and testing folds. A grouped isolate-level cross validation (i.e. leave-one-isolate-out validation) was not indicated because several serotypes were represented by only one or two isolates. The two 1–14 isolates were not included in the training set, as their precise serotype is not known, and the FT-IR exploratory data analysis showed that they exhibited completely different spectral profiles.

## Results

### Exploratory data analysis

**Figure 1:**
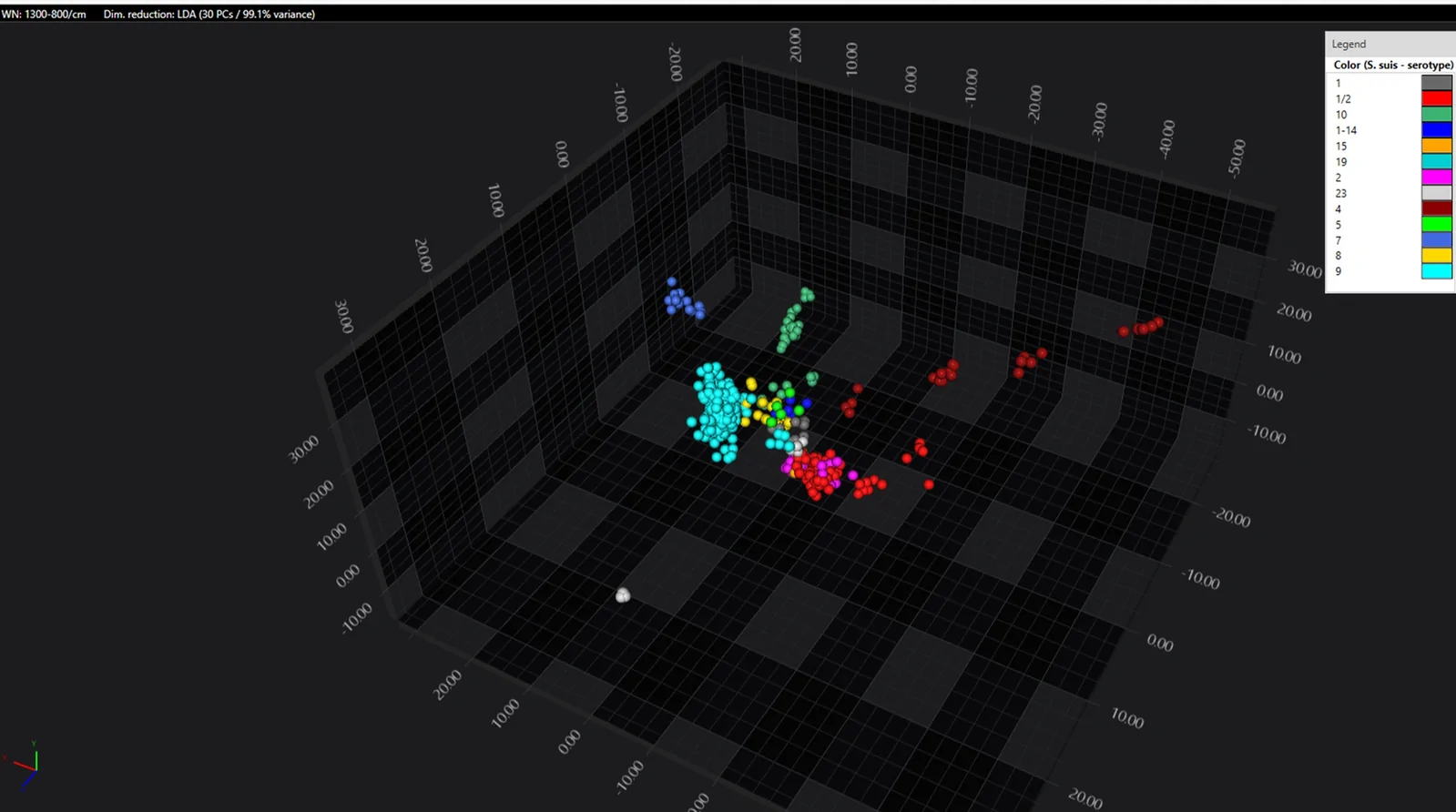
**3D PCA/LDA scatterplot showing the IR Biotyper clustering of *S. suis* sero types**. Each geometrical form represents a spectrum (all replicates were plotted), with colour indicating different serotypes. LDA was performed using 30 principal components, which covers a variance of 99.1%.

The examination of the full dataset in the 1300–800 cm^−1^ spectral region revealed the highest discriminatory power for distinguishing *S. suis* serotypes. Indeed, PCA/LDA three-dimensional (3D) scatterplot analysis shows a well-defined separation of serotypes 4, 7, 9, 10, and 23 (Figure 1).

Separation of serotypes 1, 1/2, 2, 5, 8, 1–14, 15, and 19 is visible in the deviation plot, where all the dimensions used for the discrimination of IR classes in the multidimensional space can be observed (Additional file 1).

Each group is discriminable from the others in at least one dimension, with the exception of serotypes 1/2 and 2, which are indistinguishable, and serotypes 1 and 1–14 (Additional files 2, 3).

### Machine learning

**Figure 2:**
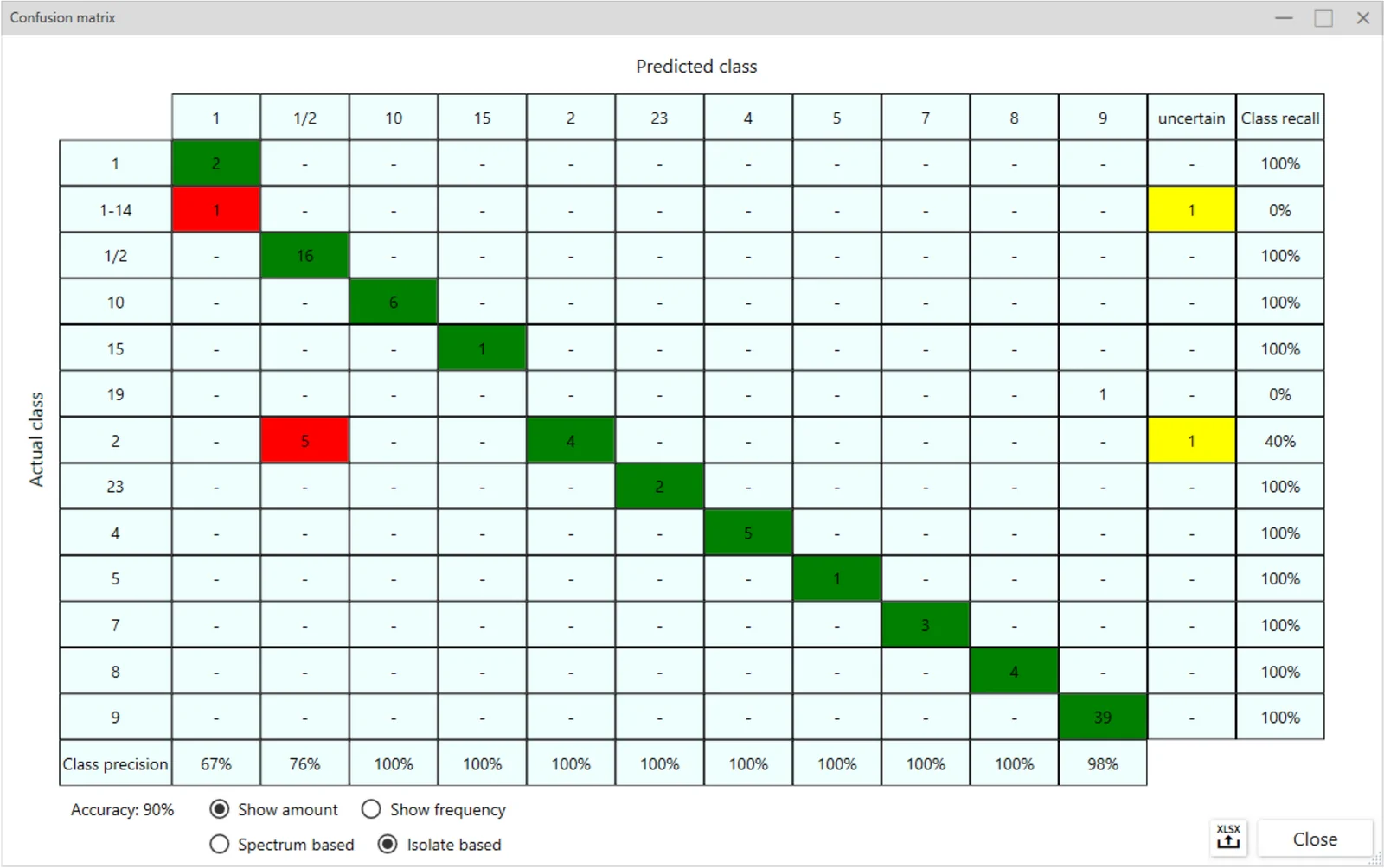
**Performance of the RBF SVM classifier**. The numbers in the box correspond to the number of isolates.

The machine learning algorithm which showed the best performance in the internal validation was the RBF SVM, with C = 10 and gamma = 0.0001 (Figure 2). The classifier showed accuracy of 90% when considering serotypes 2 and 1/2 as separate classes (83/92 isolates), with five serotype 2 isolates classified as 1/2, one serotype 2 classified as uncertain, and the single serotype 19 isolate classified as serotype 9. Of the two isolates 1–14, one was classified as serotype 1 and the other as uncertain. The classifier achieved accuracy of 96.7% (89/92 isolates), considering serotypes 2 and 1/2 as a unique class (one misclassification and two uncertain). The increase in accuracy obtained with the combined 2–1/2 category mainly reflects the resolution of reciprocal misclassification between these two spectrally overlapping serotypes and should therefore not be interpreted as an improved ability of FT-IR spectroscopy to discriminate serotypes 2 and 1/2 individually.

## Discussion

In the present study, FT-IR spectroscopy proved to be a very promising and reliable phenotypic tool for the discrimination of *S. suis* serotypes. The ability to accurately differentiate serotypes using a rapid, culture-based, and cost-effective approach could represent a significant advance for both diagnostic microbiology and epidemiological surveillance, particularly given the zoonotic relevance and genetic heterogeneity of *S. suis*.

FT-IR spectroscopy captures the global biochemical fingerprint of bacterial cells, reflecting variations in carbohydrates, proteins, lipids, and nucleic acids. In *S. suis*, capsular polysaccharides represent the major serotype-defining structures, and their biochemical composition is known to vary substantially among serotypes. These structural differences are expected to strongly influence infrared absorption patterns, particularly in the carbohydrate-dominated spectral region (1200–900 cm^−1^), which is mainly associated with polysaccharide components of the bacterial cell envelope and has been shown to provide discriminatory information for capsular serotypes [29]. Our results confirm that this spectral region contributes substantially to serotype discrimination, in agreement with previous FT-IR-based bacterial typing studies targeting surface polysaccharides and cell envelope structures [16, 20].

The possibility of successful FT-IR discrimination of *S. suis* isolates according to serotype is particularly relevant in light of the limitations associated with conventional serotyping methods. Classical serotyping based on PCR detection of capsular polysaccharide synthesis genes is susceptible to cross-reactions and often fails to classify non-typeable or novel variants [8, 30]. Whole-genome sequencing provides high-resolution typing but remains costly and time-consuming for routine diagnostics. In this context, FT-IR represents a powerful complementary approach that combines speed, reproducibility, and discriminatory power, making it well suited for high-throughput screening.

Our findings are consistent with previous studies demonstrating the capability of FT-IR spectroscopy to discriminate bacterial taxa at the subspecies or serotype level. To the best of our knowledge, no studies have previously evaluated FT-IR spectroscopy for the discrimination of *S. suis* serotypes. Therefore, the present study provides the first evaluation of FT-IR for *S. suis* serotyping, assessing its ability to discriminate multiple serotypes and its potential for supervised machine learning classification. FT-IR spectroscopy has been successfully applied to differentiate serotypes of *Salmonella enterica*, *Listeria monocytogenes*, *Escherichia coli*, and *Streptococcus pneumoniae*, often showing strong concordance with molecular typing methods [23, 24, 31, 32]. In *S. suis*, where capsule composition is a major determinant of virulence and host specificity, the biochemical sensitivity of FT-IR spectroscopy offers a clear advantage over purely genetic methods that may not fully reflect phenotypic variation.

The use of multivariate statistical approaches and machine learning algorithms further enhances the discriminatory capacity of the FT-IR data. Similar to what has been demonstrated for other bacterial species, supervised classification models, particularly support vector machines, showed promising high accuracy in assigning isolates to the correct serotype. When serotypes 2 and 1/2 were considered as separate categories, the classifier achieved overall accuracy of 90%. The increase to 96.7% observed when these two serotypes were combined into a single category should be interpreted cautiously, as it mainly results from converting the reciprocal misclassification between serotypes 2 and 1/2 into correct classifications. Thus, higher accuracy reflects the performance of the classifier under a combined 2/1/2 classification scheme rather than an ability of FT-IR spectroscopy to discriminate these two serotypes individually. This finding is consistent with the substantial spectral overlap observed between serotypes 2 and 1/2 and with their highly similar capsular polysaccharide (CPS) structures. Overall, these results confirm that FT-IR spectral data contain sufficient discriminatory information to support automated classification systems, potentially enabling routine implementation in diagnostic laboratories without advanced bioinformatics infrastructure.

From an epidemiological perspective, the ability to rapidly distinguish *S. suis* serotypes is of major importance. Certain serotypes, particularly serotypes 2 and 9, are strongly associated with invasive disease in both swine and humans, while others are more frequently linked to asymptomatic carriers. Rapid serotype identification is therefore essential for outbreak investigations, the selection of the appropriate vaccine, surveillance of zoonotic risk, and monitoring of emerging strains. Compared with molecular approaches, FT-IR spectroscopy offers a faster turnaround time and lower operational costs, making it particularly suitable for large-scale surveillance and real-time outbreak screening.

Nevertheless, some limitations should be acknowledged. As with other phenotypic methods, FT-IR performance may be influenced by culture conditions, sample preparation, and biological variability among strains. Moreover, closely related serotypes with minimal capsular differences may occasionally show partial spectral overlap. This is particularly relevant for serotypes 2 and 1/2, whose capsular polysaccharides are structurally highly similar and differ only by the substitution of galactose (Gal) in serotype 2 with *N*-acetylgalactosamine (GalNAc) in the CPS side chain of serotype 1/2 [33, 34]. This close structural similarity may account for the partial overlap observed in their FT-IR spectral profiles and the greater difficulty in discriminating against these two serotypes. Expanding reference libraries and increasing the number of well-characterized isolates will be essential to further improving classification robustness, as well as extensive validation with field samples. Integration of FT-IR with genomic data could also provide a powerful combined approach, leveraging the speed of spectroscopy with the resolution of sequencing.

In conclusion, this study demonstrates that FT-IR spectroscopy could represent a novel reliable, rapid, and cost-effective tool for the discrimination of *S. suis* serotypes. Its promising performance, ease of use, and compatibility with machine learning approaches support its potential applicability for implementation in routine diagnostic and epidemiological workflows. As already observed for other clinically relevant bacteria, FT-IR spectroscopy could represent a valuable complementary method to molecular typing, particularly in settings where rapid decision-making and high-throughput analysis are required.

## Supplementary Information

**Additional file 1. Deviation plot showing the separation of all the** ***S. suis*** **in the multidimensional IR spectral space.**

**Additional file 2. Deviation plot showing serotypes 2 and 1/2**.

**Additional file 3. Deviation plot showing serotypes 1 and 1–14.**

## Data Availability

No datasets were generated or analysed during the current study.
